# Radiotherapy for non-malignant disorders: state of the art and update of the evidence-based practice guidelines

**DOI:** 10.1259/bjr.20150080

**Published:** 2015-06-12

**Authors:** M H Seegenschmiedt, O Micke, R Muecke, the German Cooperative Group on Radiotherapy for Non-malignant Diseases (GCG-BD)

**Affiliations:** ^1^ Center for Radiotherapy and Radiation Oncology, Strahlenzentrum Hamburg, Hamburg, Germany; ^2^ Department of Radiotherapy and Radiation Oncology, Franziskus Hospital Bielefeld, Bielefeld, Germany; ^3^ Department of Radiotherapy, Lippe Hospital Lemgo, Lemgo, Germany; ^4^ Department of Radiotherapy and Radiation Oncology, Marien Hospital Herne, Ruhr University Bochum, Bochum, Germany

## Abstract

Every year in Germany about 50,000 patients are referred and treated by radiotherapy (RT) for “non-malignant disorders”. This highly successful treatment is applied only for specific indications such as preservation or recovery of the quality of life by means of pain reduction or resolution and/or an improvement of formerly impaired physical body function owing to specific disease-related symptoms. Since 1995, German radiation oncologists have treated non-malignant disorders according to national consensus guidelines; these guidelines were updated and further developed over 3 years by implementation of a systematic consensus process to achieve national upgraded and accepted S2e clinical practice guidelines. Throughout this process, international standards of evaluation were implemented. This review summarizes most of the generally accepted indications for the application of RT for non-malignant diseases and presents the special treatment concepts. The following disease groups are addressed: painful degenerative skeletal disorders, hyperproliferative disorders and symptomatic functional disorders. These state of the art guidelines may serve as a platform for daily clinical work; they provide a new starting point for quality assessment, future clinical research, including the design of prospective clinical trials, and outcome research in the underrepresented and less appreciated field of RT for non-malignant disorders.

Every year about 50,000 patients in Germany are treated for “non-malignant disorders” respectively “benign disease conditions” by using ionizing radiation applied in >300 radiotherapy (RT) facilities.^1–4^ The aim of these treatments are and will be the preservation or recovery of various quality of life aspects, for example, by prevention of or reduction of pain and/or improvement of formerly disabled physical body functions.

Non-malignant indications for RT comprise about 10–30% of all treated patients in most academic, public and private RT facilities in Germany. Over the past decade, various so called patterns of care studies (PCSs) have focused on the general and various specific aspects of these diseases and their RT treatment conditions and concepts in Germany.^1–5^ Overall, there is not a single RT institution among all 300 active RT facilities in Germany that does not offer RT for these benign or “non-malignant diseases”.^1–4^

Since 1995 and together with the foundation of the German Society of Radiation Therapy and Oncology (DEGRO), a scientific task force group was formed, the German Cooperative Group on Radiotherapy for Benign Diseases (GCG-BD), which undertook the task to review the large amount of clinical experience gained in several decades from 1930 to 1990 in Germany about the use of RT for non-malignant disorders; the relevant articles and clinical data were systematically discussed and evaluated by a scientific panel and a “Delphi” consensus process involving all active RT providers. The first National guideline was defined and published in the year 2000.^1^ From then on, specific PCSs and prospective randomized clinical trials were developed to improve the available levels of evidence (LOEs) for various non-malignant disorders.^5–8^ Meanwhile, a considerable number of clinical trials have been carried out and published.^9–14^

The updated National practice guideline v. 2.0 of the most common RT indications for non-malignant diseases were developed between 2010 and 2013 by a nominated group of specialists in conjunction with all members of the German Radiation Oncology Society (DEGRO) and GCG-BD; the Delphi consensus process comprised several national-held symposia, working group meetings and the circulation of all preliminary text versions within the responsible writing committee group members and the final presentation in the national scientific DEGRO meeting in the year 2013.

These updated practice guidelines focus on those clearly defined RT indications that have become clinically relevant in terms of the high clinical demand (*i.e.* number of referrals from other medical disciplines), and the currently achieved quantity and quality of treatments, which had been determined by an evaluation of the continuously increasing number of treated patients between the first two evaluation periods within Germany (Table 1).

**Table 1.** Development of radiotherapy for non-malignant diseases in Germany (number of treated patients from 1999 to 2004—results of patterns of care studies)

Non-malignant diseases (treatment groups)	1999	2004	Increase (%)
Inflammatory	456	503	10.9
Degenerative	12,600	23,754	88.5
Hyperproliferative	972	1252	28.8
Functional/other	6099	10,637	74.4
Overall	20,082	37,410	86.3

The largest group of patients with non-malignant disorders and indications for the use of RT are those suffering from painful degenerative joint disorders.^1–3,5^

There are several general rules established in the field of RT for non-malignant disorders: (1) RT of non-malignant conditions is usually carried out with much shorter time schedules and with much lower single and total RT doses than those applied for malignant tumours; however, the responsibilities of the involved radiation oncologists and therapists with regard to quality and delivery of RT treatment are the same as those for malignant disorders; (2) careful preparation, diligent performance and complete documentation of all RT treatments for non-malignant disorders are mandatory; (3) long-term follow-up evaluation of the whole treatment process has to be performed with utmost care and attention, as it is the case with any patient suffering from a malignant disorder.

With regard to the specific national medical and jurisdictional background and the different justifications in Germany and in other European countries, there is a special need for updated and established national treatment guidelines for RT of non-malignant diseases similar to those already developed for most malignant disorders.^5,6,15,16^ Additionally, the European Society of Therapeutic Radiation and Oncology has performed two symposia in the past (Brussels, 1999, and Nice, 2005) on this issue, which resulted in general recommendations for the different practitioners in European countries.^17^

## METHODS AND MATERIALS

In the first step, the available data from the literature (MEDLINE, PubMed, Cochrane) and pertinent information from clinicians and patient groups were systematically reviewed, and the relevant key articles, usually with high scientific impact, were identified. The LOEs for each disease entity were determined and finally graded according to the well-known international recommendations (Tables 2 and 3).^18^

**Table 2.** Recommended treatment energies in relation to the selected depth for the reference point of the treated target volume

Treatment unit	Energy	Reference depth
X-ray therapy unit—superficial	10–50 keV	Surface
X-ray therapy unit—low depth	50–100 keV	<2 cm
X-ray therapy unit—orthovoltage	100–400 keV	<5 cm
Cobalt radiation units	1.17 bzw. 1.33 MeV	<10 cm
Linear accelerator		
Photons	6–18 MeV	All depths use of bolus material if necessary
Electrons	6–21 MeV	
Brachytherapy (Strontium-90 source)	2.2 MeV β^−^radiation Contact treatment	<10 mm

**Table 3.** Diseases, studies, numbers of patients, highest levels of evidence, and radiotherapy indications and recommendations

Specific disease	Number of studies	Number of evaluated patients	Highest level of evidence	Highest level of recommendation	Main indication group
Painful arthrosis of the knee joint	23	10,046	2c (PCS)	B (shall be performed)	More than 3 months of inflammatory signs, not responding to other therapeutic measures
Painful arthrosis of the hip joint	19	895	4 (ReS)	C (might be performed)	More than 3 months of inflammatory signs, not responding to other therapeutic measures
Painful arthrosis of the hand and finger joints	17	809	4 (ReS)	C (might be performed)	More than 3 months of inflammatory signs, not responding to other therapeutic measures
Painful shoulder syndrome	17	8240	2b (DOS)	B (shall be performed)	More than 3 months of inflammatory signs, not responding to other therapeutic measures
Painful elbow syndrome	22	2141	2b (DOS)	B (shall be performed)	More than 3 months of inflammatory signs, not responding to other therapeutic measures
Painful trochanteric bursitis	2	60	4 (ReS)	C (might be performed)	More than 3 months of inflammatory signs, not responding to other therapeutic measures
Painful plantar fasciitis	22	11,909	1b (RS)	A (should be performed)	More than 3 months of inflammatory signs, not responding to other therapeutic measures
Morbus Dupuytren	12	1762	2b (DOS)	B (shall be performed)	Early nodular stage
Morbus Ledderhose	6	200	4 (ReS)	C (might be performed)	Painful detectable or palpable lesions
Keloids	13	4317	2c (PCS)	B (shall be performed)	Affected palpable lesions after surgical excision
Peyronie's disease	20	8732	2c (PCS)	B (shall be performed)	Soft localized penile plaques
Desmoid tumours	40	2238	2c (ReS)	B (shall be performed)	Complete inclusion of the involved structures
Symptomatic vertebral haemangiomas	66	548	2c (PCS)	B (shall be performed)	Painful vertebral haemangiomas
Pigmented villonodular synovitis	20	195	2c (PCS)	B (shall be performed)	Affected synovial cells
Gorham Stout syndrome	39	62	2c (PCS)	B (shall be performed)	Symptomatic cases (affected bones)
Heterotopic ossification	7	8682	1b (RS)	A (should be performed)	After hip joint replacement, pre- or post-operative
Graves orbitopathy	36	2039	2b (DOS)	B (shall be performed)	Ophthalmologic symptoms of early and advanced inflammatory phase

DOS, dose optimization study; PCS, patterns of care study; ReS, retrospective study; RS, randomized study.

In the second step, the expert panel prepared a first consensus draft that was opened to propositions and comments from all participating institutions according to the established Delphi process and during two consecutive national RT conferences related to these topics.

In the third step, after completion of the consensus discussion, a final version of the updated guideline was written by the expert panel, presented and finally approved by the executive committee of the German Society of Radiation Oncology (DEGRO).

In the fourth and final step, all guidelines were presented and distributed at a national meeting of the radiation oncologists and therapists in the year 2013.

## RESULTS

The relevant disease-specific guidelines are presented in the following sections.

### Painful degenerative skeletal disorders

#### Indication, radiation technique and dose

RT is only recommended if the previous standard non-radiotherapeutic approaches have failed. Furthermore, related to the possible aspect of tumour induction, younger patients (less than 40 years) should be treated only in exceptional cases and after careful evaluation of all potential risks compared with the expected benefit.

Single doses of 0.5–1.0 Gy, total doses of 3.0–6.0 Gy and two or three fractions per week with orthovoltage or megavoltage techniques are the recommended techniques. Generally, the target volumes for the different enthesopathies, such as the painful rotator cuff syndrome of the shoulder; the tennis and golfer elbow and plantar or dorsal heel syndrome, encompass the complete involved insertion zone together with the nearby bony and muscular, and soft-tissue structures. For all types of painful arthrosis in various locations, it is necessary to include the articular cartilage, the neighbouring bone, the entire synovium as well as the joint surrounding muscles and the periarticular connective tissue. In case of persisting pain or insufficient pain relief 6–12 weeks after the first RT series, a second RT series may be recommended.^15^

For all painful degenerative disorders, it is required to obtain at least conventional X-rays, which should be obtained in at least two standardized 90° planes to exclude accidental neoplastic processes. In unclear clinical situations, it may be necessary to evaluate additional bone scintigraphy, CT and, especially, MRI prior any indication for RT.^15,19^

From a practical standpoint, visual analogue scales (VASs; from 0 to 10) and the subjective score according to von Pannewitz^20^ (improved/no change/worsened) should be used for recording the symptomatic changes and the final outcome at least 6–8 weeks after completion of RT.

The recommended physical aspects and relevant beam energies are given in relation to the selected depth for the reference point for all non-malignant disorders (Table 2).

#### Painful arthrosis of the knee joint—results following radiotherapy

Within the past 20 years, a total of 23 clinical studies (22 retrospective studies and 1 PCS) have been conducted. Moreover, the clinical outcome results of a total of 10,046 patients who had been treated with low-dose RT for painful arthrosis of the knee joint were evaluated and published; 5069 of these patients were evaluated within the German PCS in 2010. Overall, a markedly and complete pain reduction was shown in 58–91% of all irradiated patients.^4^

#### Painful arthrosis of the hip joint—results following radiotherapy

A total of 19 retrospective clinical studies were evaluated, and the results of 895 patients who had been treated with low-dose RT for painful arthrosis of the hip joint were evaluated and published. A marked and complete pain reduction was shown in 24–89% of these irradiated patients.^15^

#### Painful arthrosis of the hand and finger joints—results following radiotherapy

A total of 17 retrospective clinical studies were evaluated, and the results of 809 patients who had received low-dose RT for painful arthrosis of the hand and finger joints were evaluated and published. A marked and complete pain reduction was shown in 63–75% of irradiated patients.^21–23^

#### Painful shoulder syndrome—results following radiotherapy

A total of 17 clinical studies (16 retrospective studies and 1 dose optimization study) were evaluated, and the results of 8240 patients who had been treated with low-dose RT for painful shoulder syndrome were evaluated and published. The range of complete and partial pain response was between 58% and 100% of all irradiated patients at follow-up 2–3 months after completion of RT.^11,24,25^ The indication of an earlier start of RT with less than 6 months after the onset of the pain symptoms was shown to be more effective than with a more chronic pain syndrome of 1 year and longer. The clinical outcome data for patients with calcifications were inconsistent and did not show higher success rates in all clinical studies.^15^

#### Painful elbow syndrome—results following radiotherapy

A total of 22 clinical studies (21 retrospective studies and 1 dose optimization study) were evaluated, and the results of 2141 patients who had been treated with low-dose RT for painful elbow syndrome were evaluated and published. A partial and complete pain reduction was demonstrated in 63–75% of irradiated patients.^12,15,26^

#### Painful trochanteric bursitis—results following radiotherapy

So far only 2 retrospective clinical studies were published summarizing the outcome of 60 patients who had been treated with low-dose RT for painful trochanteric bursitis. A partial and complete pain reduction was shown in 56–73% of irradiated patients.^27,28^

#### Painful plantar fasciitis—results following radiotherapy

A total of 22 clinical studies (18 retrospective studies, 2 dose optimization studies, 1 PCS and 1 randomized study) were evaluated, and the results of 11,909 patients who had been treated with low-dose RT for painful plantar fasciitis were published. Overall, the range of complete pain relief (“CR”) reached 12–81%, and the partial pain relief (“PR”) ranged from 7% to 74% in the published studies.^9,10,13,15,29–31^

The dose optimization trial was designed to compare 2 dose regimens: 3.0 Gy with 6 fractions of 0.5 Gy *vs* 6.0 Gy with 6 fractions of 1.0 Gy in 587 patients. The use of RT led to a highly significant reduction of pain symptoms in both groups; however, the lower dose regimen was equally effective as the higher dose regimen.^10,13,31^ In another randomized clinical trial, the efficacy of two other dose concepts was evaluated in a total of 66 patients who had been treated with either 6.0 Gy with 6 fractions of 1.0 Gy or with 0.6 Gy with 6 fractions of 0.1 Gy. After 1 year follow-up, the higher-dose group had obtained a highly significantly better pain control than the very low-dose group.^9^

#### Treatment response evaluation

Most clinical trials evaluate the treatment response related to “pain relief” and/or “freedom of pain” at follow-up periods in the range of 2–3 months after RT; not all clinical studies obtain response rates beyond 1-year follow-up to evaluate possible delayed response effects. For the daily clinical work, both the VASs (from 0 to 10) and the score according to von Pannewitz^20^ (improved/no change/worsened) should be used for recording of symptomatic outcome.^20^

#### Possible placebo effect

In previously published double-blinded studies from the 1970s, which did not fulfil current quality criteria of prospective clinical trials, a variety of different degenerative skeletal diseases were treated with low-dose RT. In contrast to the reported data from all studies above, these studies could not prove a significantly higher response for the RT group in comparison to the placebo group.^32–34^ Only Goldie et al^32^ showed a borderline significant advantage for RT for patients with painful arthrosis of the knee joint.^32^

### Hyperproliferative disorders

#### Morbus Dupuytren—indication, radiation technique and dose

The clinical implementation and efficacy of RT in morbus Dupuytren is strongly stage dependent, because the antiproliferative effect is most pronounced in the early nodular stage owing to the biological activity on the mesenchymal target tissues. In this disease stage, ionizing radiation can induce a significant reduction in size of the lesions and a reduction of the proliferation rate of the involved proliferating fibroblasts and myofibroblasts.

The target volume should always comprise the palpable nodules and cords with a minimum safety margin of 10 mm in all directions. RT can be applied either with low energetic electron beam irradiation (4–6 MeV), as well as with low X-ray energies (100–150 kV). Shielding of the uninvolved surrounding skin and soft-tissue structures by use of individual lead material should always take place. Single doses of 2–3 Gy and total doses of 15–21 Gy, and five fractions per week are recommended using one RT series.

The best clinical outcome was reported with a treatment schedule and a total dose of 30 Gy which is applied in two separate RT series of each 5 × 3 Gy; the interval between the two RT series should be in the range of 12 weeks.^35–39^

#### Morbus Dupuytren—clinical results following radiotherapy

A total of 12 clinical studies (10 retrospective studies and 2 dose optimization studies) were published, and the results of 1762 patients were evaluated. In one controlled dose optimization trial, after a long-term follow-up of 10 years using the above specified single and total doses, 84% of patients in the N stage (only nodules and cords) and 67% of N/I stage patients (extension deficit up to 1–10°) successfully avoided disease progression and did not undergo hand surgery. By contrast, in the more advanced Stages II, III and more, significantly less successful response rates were noted.^38^ In the other randomized dose optimization trial, the efficacy of two dose concepts were evaluated: patients were treated either with a single RT course of 21 Gy (7 × 3.0 Gy), while the other group received a total dose of 30 Gy applied in two series of 5 × 3.0 Gy, repeated at intervals of 12 weeks; furthermore, an untreated control group was observed during the same recruitment period. After a mean follow-up of 8 years, the disease progressed (*i.e.* increasing extension deficit) in 35% of the control group, in 7% in the 21 Gy group and only 4% in the 30 Gy group.^39^

#### Morbus Ledderhose—indication, radiation technique and dose

The radiobiological rationale is in analogy to Dupuytren's disease. Similarly, the target volume should comprise the palpable lesions with a minimum safety margin of 10 mm in all directions. The RT can be applied with low energetic electron beam irradiation, as well as lower energy X-ray irradiation. Individual lead shielding of the surrounding normal soft tissues using individualized cut-outs should be implemented. The best reported clinical outcome was achieved when using RT concepts with single doses of 2–3 Gy and total doses of 15–21 Gy per RT series, and five fractions per week; in the largest clinical study, two RT series with each 15 Gy were applied with a 12-week break between both RT series and a total dose of 30 Gy.^40^

#### Morbus Ledderhose—results following radiotherapy

A total of 6 retrospective clinical studies were published, and the results of 200 patients were analysed and evaluated. So far, only a few data are available with results for the primary RT treatment in morbus Ledderhose. 1 study analysed the results of 25 patients (36 feet) following 2 RT courses of 5 × 3.0 Gy for a total dose of 30.0 Gy. With a median follow-up of 38 months (range, 12–67 months), disease progression was prevented in all patients. Overall, 28 of 36 irradiated feet responded with a regression of pain and tenderness, and in 8 of 36 feet, the symptoms were stabilized.^40^ A further study reported results in 24 patients (33 feet), which were irradiated with 2 RT courses of 5 × 3.0 Gy for a total dose of 30.0 Gy (*n* = 20) or 2 single fractions of 4.0 Gy on consecutive days, repeated at intervals of 4 weeks to cumulative doses ranging from 24 to 32 Gy (*n* = 4). With a median follow-up of 22.5 months (range, 6–76 months), none of the patients showed a clinical progression of the lesions concerning the number, size and subjective clinical symptoms.^41^

#### Keloids—indication, radiation technique and dose

The radiobiological rationale relies on the prevention of a renewed proliferation of the mesenchymal target tissues that form after any type of trauma, including intended surgical procedures. Thus, the target volume should always comprise the initially affected visible and traumatized lesions after surgery and should include a safety margin of 10–20 mm. The RT can be applied either with low-energy X-rays (150–200 kV) or with low-energy electrons (4–10 MeV) or by means of brachytherapy. Single doses of 2–5 Gy and total doses of 16–20 Gy per series applied with four to five fractions per week are recommended. To obtain the optimal antiproliferative effect radiation therapy should be initiated immediately after the surgical excision, for example, preferably within the first 24 h. Therefore, the scar tapes and patch fixation should be left unchanged to avoid any mechanical dehiscence of the wound margins.^42,43^ Careful wound care and avoidance of scar tension within 4–6 weeks after surgery should be implemented.

#### Keloids—results following radiotherapy

A total of 13 clinical studies (12 retrospective studies and 1 PCS) were published, and the results of 4317 patients analysed. Effective treatments resulted in the avoidance of renewed excessive scar formation and good cosmesis with documented patient satisfaction. With regard to the prevention and reduction of relapse rates after excision and irradiation of keloids, the reported results ranged from 60% to 80%.^42,43^

#### Peyronie's disease—indication, radiation technique and dose

The use of RT is clinically indicated in patients with early stage disease when presenting with soft localized penile plaques; the use of radiation therapy for calcified plaques is usually associated with poor clinical results. Depending on the location of the plaques, the RT can be given either with low-energy X-rays, low-energy photons or low-energy electrons. In order to achieve a better dosage in the surface of the penile plaques, which are often located directly underneath the skin, bolus material should be implemented. The target volume should comprise the “plaque lesion” plus a safety margin of 10 mm and should be limited by individual lead collimators. Careful protection of the testes, pubic hair and the penile gland should be applied. Different set-ups can be applied for the radiation procedure: the penis can be either irradiated in an anterior/posterior projection with the scrotum and testes protected by lead shielding or with lateral opposing fields using an upright fixation technique. Single doses of 2–3 Gy and total doses of 10–20 Gy with five fractions per week are recommended.^44^

#### Peyronie's disease—results following radiotherapy

A total of 21 clinical studies (18 retrospective studies and 3 PCSs) have been published, and the results of 8732 patients were analysed and evaluated: the reported response rates and rates of pain relief ranged between 50% and 90%; in addition, an improvement of the penile deviation or curvature was obtained in 30–70%.^2,44–46^ A few clinical studies could demonstrate a softening of the penile plaques following radiation. So far, randomized clinical trials comparing RT with placebo treatment or other established treatments are missing.

#### Desmoid tumours/aggressive fibromatosis

Desmoid tumours also termed aggressive fibromatosis are benign tumour formations, which have a high local relapse rate after local surgery. Radiation therapy is usually applied in advanced disease situations when surgery is not possible or has failed to achieve local tumour control. Adjuvant RT is applied in high-risk situations when the resection margin is close or microscopically positive (R1-resection).

#### Indication, radiation technique and dose

In order to define the exact target volume, the use of CT and MRI and the fusion of both imaging procedures is highly recommended for three-dimensional treatment planning. The definition of the target volume definition should be in accordance with the guidelines for sarcomas. In the adjuvant setting following surgical resection, single RT doses of 1.8–2.0 Gy with five weekly fractions and a total RT dose of 50–60 Gy should be applied. In the primary setting without surgery or in case of macroscopic disease or local relapse, increased total RT doses of 60–65 Gy are recommended.

#### Desmoid tumours/aggressive fibromatosis—results following radiotherapy

A total of 36 retrospective case series, 2 meta-analyses, 1 prospective Phase 2 clinical study and 1 PCS have been published, and the results of 2238 patients were analysed and evaluated. Unfortunately, the results from prospective randomized trials are still not available.^47^ 1 meta-analysis including the results of 22 studies revealed no significant difference in outcome between the primary RT without surgery and the adjuvant postsurgical RT; nevertheless, the local control rate was superior with the use of RT compared with local resection alone.^48^ Another meta-analysis including a total of 698 patients from 13 clinical studies revealed that adjuvant RT after R0 resection improved the local control rate by 17%. In addition, prognostic factors such as tumour size seemed not to have any significance for the achievement of local control.^49^ The recently published prospective EORTC study showed that RT with 56 Gy is effective for patients with inoperable progressive disease of primary, recurrent or incompletely resected lesions. Although the clinical response pattern following irradiation was slow, in the long-term analysis over periods of 3 years and more, the regression rate increased continuously.^50^

In summary, both settings, the primary RT as well as post-operative RT have been shown to achieve a high rate of local control.

#### Symptomatic vertebral haemangiomas—indication, radiation technique and dose

The rationale and treatment goals for the implementation of RT are the achievement of pain control, improvement of neurological deficits and prevention of further local progression. Possible radiobiological mechanisms discussed are the direct inhibition of proliferation of the endothelial cells and the obliteration of afferent vessels within the haemangioma. From retrospective clinical studies and using a statistical logistic regression analysis, a significant higher rate of symptom relief and local control was achieved when total RT doses of >34 Gy had been applied. This effect has been confirmed by other retrospective data that showed a significantly improved control rate in patients receiving total RT doses of ≥36 Gy. Usually, single RT doses of 1.8–2.0 Gy using five fractions per week are recommended.^51–53^

#### Symptomatic vertebral haemangiomas—results following radiotherapy

A total of 65 retrospective case series and 1 PCS have been published, and the results of 548 patients have been analysed and evaluated. The retrospective data summarize the outcome of 464 cases: complete pain relief was achieved in 57.6% and partial pain relief in 27.7% of all treated cases, while 14.7% remained without symptomatic improvement. Thus, an overall response rate following RT could be achieved in 85.3%.^51^

The German PCSs comprised 84 patients with a total of 96 symptomatic lesions. After a median follow-up of 68 months (range, 6–422 months), the overall response rate was 90.5%. Complete symptomatic remission of symptoms occurred in 61.9% of all irradiated lesions, and 28.6% obtained a partial remission of symptoms, while 9.5% achieved no pain relief at all. More interestingly, radiological signs of remineralization of irradiated bones were noted in 26.2% of all cases.^52^

#### Pigmented villonodular synovitis—indication, radiation technique and dose

The rationale and goal of RT is to reduce the proliferation of abnormal synovial cells, which can destroy the cartilage, bony and soft-tissue structures of any affected joint. Three forms have to be differentiated: the more aggressive diffuse type [D-pigmented villonodular synovitis (D-PVNS)], the more benign localized type (L-PNVS) and the pigmented villonodular tenosynovitis (PVTS). The indication for the use of RT is either an additive treatment after incomplete surgery or a salvage treatment in case of renewed progression after failing one or more previous resection procedures. Thus, the target volume should include the primary extension of the lesion(s) together with the entire synovial lining layers of the involved joint and a sufficient safety margin of 10–20 mm in all directions. To achieve a complete coverage of the affected “risk regions”, a CT- and MRI-based treatment planning including an image fusion of both imaging devices is mandatory. With regard to RT concept, single RT doses of 1.8–2.0 Gy with five fractions per week are recommended; in case of a diffuse type (D-PVNS) lesion, total RT doses should reach 36–40 Gy, whereas in cases of a localized type (L-PNVS) and of PVTS total RT doses of 30–36 Gy are considered sufficient.^54–56^

#### Pigmented villonodular synovitis—results following radiotherapy

A total of 19 retrospective case series and 1 PCS have been published, and the results of 195 irradiated lesions were evaluated and analysed.

Overall, the retrospective case series (*n* = 154) could demonstrate a local control rate of up to 100% when using total RT doses of 20–50 Gy.^54^ The German PCS (*n* = 41) yielded a local control rate of 95% with total RT doses of 20–50 Gy.^55^

#### Gorham Stout syndrome—indication, radiation technique and dose

The disease is characterized by a progressive bony destruction with subsequent loss of stability and localized pain. Ionizing radiation can stop the progression of the disease and should be performed in symptomatic cases. Single RT doses of 1.8–2 Gy and total RT doses of 36–45 Gy with five fractions per week are recommended. The additional administration of bisphosphonates over several months may support and increase the rate of remineralization of bony structures.

#### Gorham Stout syndrome—results following radiotherapy

A total of 38 retrospective case series and 1 PCS have been published, and the results of 62 patients were evaluated and analysed. With follow-up periods ranging from 2 to 288 months, the retrospective case series could demonstrate the achievement of local control in up to 75–77%.^57,58^ With a median follow-up of 42 months, the German PCS (*n* = 10) revealed that local progression could be avoided in 80% of cases when using total RT doses of 36–45 Gy; moreover, 27% showed signs of bony remineralization after RT.^57^

### Symptomatic functional disorders

#### Heterotopic ossification—indication, radiation technique and dose

Heterotopic ossification (HO) is a pathological repair process, which is characterized by excessive bone formation in soft-tissue structures about joints after fractures and other traumatic events, including the most intended surgical procedures such as total hip arthroplasty, and other joint replacement procedures. In high-risk situations of hip joint replacement, the use of either pre-operative or post-operative RT of the hip region provides an effective prophylactic means for reducing the risk of HO. To achieve an optimal outcome, RT should be applied during a radiosensitive period of the bony precursor cells. Thus, a relatively narrow time window of 4 h before and up to 72 h after surgery has to be respected for the application of RT.^59^ In accordance with the reported outcome of several controlled clinical studies, both pre- and post-operative RT can be carried out once with a single dose of 7–8 Gy. Only for patients with more pronounced risk factors such as severe ossification Brooker grade III–IV post-operative fractionated RT is preferred.^60^ In patients with major risk factors, post-operative RT is applied with five daily fractions of 3.5 Gy up to a total dose of 17.5 Gy.^16,59,61–65^

The target volume of prophylactic RT treatment should comprise the whole joint and the typical localizations of heterotopic bone formation with a safety margin of 10–20 mm.^16,66^ Potential risk structures such as the bladder, rectum and small intestine should be protected by lead shielding devices. RT should always be applied using high energy linear accelerator photons. Other articular regions such as the shoulder, elbow or knee should be treated similar to the approach with hip joint replacement.

#### Heterotopic ossification—results following radiotherapy

Among the numerous published retrospective clinical studies, a total of seven studies (one prospective study, two retrospective studies, one PCS, two randomized study and one meta-analysis) are of high practical value. The results of 8682 patients treated with RT for the prevention of HO were evaluated and analysed. These studies revealed a high rate of prevention of HO after hip joint replacement with up to 90% by means of pre- or post-operative RT.^16,59,60,62–65^

#### Graves orbitopathy—indication, radiation technique and dose

Graves' orbitopathy (GO) is mostly caused by an overactivity of the thyroid gland (hyperthyroidism) and is associated with the swelling of the eye muscles and inflammatory or fibrotic changes of the retro-orbital soft tissue, creating a bulging of the involved eyes. The clinical use of RT in the multidisciplinary treatment of GO is still a matter of controversial discussion. In Germany, like in most European countries, RT is applied in specific disease categories, which comprise dysfunction of the eye muscles and are defined as Categories 3–5 according to the NOSPECS classification.^16,67–69^ The radiobiological rationale for the use of RT is justified by the proven anti-inflammatory and antiproliferative effects on the involved tissues that induces a remission of the inflammatory changes and stops the fibrotic hyperproliferation; thereby the inflammatory phase can be shortened, eye muscle dysfunctions can be resolved and possible late complications such as optic nerve entrapment, loss of vision or double vision can be avoided.^70^ Prior to the start of RT, a normal thyroid metabolic status should be achieved by appropriate medication.

RT of GO should be carried out with lateral opposing 4–6 MV photon beams of a linear accelerator and using a head mask fixation device. The target volume should be defined by using CT-based three-dimensional treatment planning and possibly with additional fusion of MRI scans. The margins of the target volume is defined as: dorsal margin of the orbita at the Zinn's optic nerve exit zone, ventral margin including two-thirds of ocular bulb and up to a 6-mm zone posterior to the corneal limbus and covering the insertion of all extraocular muscles.^71–73^

The RT dose concepts should be adjusted to the individual phase of the disease. In the early inflammatory phase, daily single doses of 0.3–2.0 Gy with eight fractions and total RT doses of 2.4–16.0 Gy should be prescribed. In advanced inflammatory and fibrotic phases, usually a higher single RT dose of 2 Gy with eight to ten fractions and total RT dose of 16–20 Gy are recommended. In patients with severe ophthalmologic symptoms, the efficacy of RT can be improved by using reduced single RT doses of 1 Gy and total RT doses of 20 Gy and application of RT only once per week.^16^

Currently, it is not clear whether considerably lower RT doses are equally effective, depending on the stage of the disease. Nevertheless, lower total RT doses may reduce the potential risk for radiogenic induction of secondary tumours.^74–80^

#### Graves orbitopathy—results following radiotherapy

A total of 29 retrospective case series, including 5 randomized studies and 2 dose optimization studies have been published, and the results of 2039 patients with RT for GO were analysed and evaluated. With regard to the achieved treatment response, measured by objective criteria such as remission of eye symptom, about 65–75% of the patients with GO showed good or excellent response rates after RT.^16,68,70,74,77,80,81^

### Summary—recommendations

All clinical data and the defined recommendations for application of RT in the previously described disease entities are given in Tables 3 and 4.

**Table 4.** Recommendations for single and total doses

Specific disease	Single dose (Gy)	Total dose (Gy)
Painful arthrosis of the knee joint	0.5–1.0	3.0–6.0
Painful arthrosis of the hip joint	0.5–1.0	3.0–6.0
Painful arthrosis of the hand and finger joints	0.5–1.0	3.0–6.0
Painful shoulder syndrome	0.5–1.0	3.0–6.0
Painful elbow syndrome	0.5–1.0	3.0–6.0
Painful trochanteric bursitis	0.5–1.0	3.0–6.0
Painful plantar fasciitis	0.5–1.0	3.0–6.0
Morbus Dupuytren	3.0	15.0 (repeat after 12 weeks)
Morbus Ledderhose	3.0	15.0 (repeat after 12 weeks)
Keloids	3.0	12.0
Peyronie's disease	2.0–3.0	10.0–20.0
Desmoid tumours	1.8–2.0	50.0–65.0
Symptomatic vertebral haemangiomas	1.8–2.0	34.0–36.0
Pigmented villonodular synovitis	1.8–2.0	36.0–40.0
Gorham Stout syndrome	1.8–2.0	36.0–45.0
Heterotopic ossification (pre-operative)	7.0	7.0
Heterotopic ossification (post-operative)	3.5	17.5
Graves orbitopathy (early inflammatory phase)	0.3–2.0	2.4–16.0
Graves orbitopathy (advanced inflammatory phase)	2.0	16.0–20.0

## CONCLUSION

This overview summarizes the results of the updated German Evidence-based Consensus Guidelines for RT of non-malignant diseases. These results may serve as a starting point for continuous quality assessment, future clinical research, including the design of prospective clinical trials, and outcome research for non-malignant disorders treated with RT.
